# A co-production approach guided by the behaviour change wheel to develop an intervention for reducing sedentary behaviour after stroke

**DOI:** 10.1186/s40814-020-00667-1

**Published:** 2020-08-17

**Authors:** Jennifer Hall, Sarah Morton, Jessica Hall, David J. Clarke, Claire F. Fitzsimons, Coralie English, Anne Forster, Gillian E. Mead, Rebecca Lawton

**Affiliations:** 1 Academic Unit for Ageing and Stroke Research, Leeds Institute of Health Sciences, University of Leeds, Bradford, BD9 6RJ UK; 2 Academic Unit for Ageing and Stroke Research, Bradford Institute for Health Research, Bradford Teaching Hospitals NHS Foundation Trust, Bradford, BD9 6RJ UK; 3 Centre for Clinical Brain Sciences, University of Edinburgh, EH16 4A, Edinburgh, UK; 4 Physical Activity for Health Research Centre, St Leonards Land, Holyrood Road, Edinburgh, EH8 8AQ UK; 5 School of Health Sciences and Priority Research Centre for Stroke and Brain Injury, University of Newcastle, Newcastle, Australia; 6 School of Psychology, University of Leeds, Leeds, LS2 9JT UK

**Keywords:** Co-production, Behaviour change wheel, Intervention development, Stroke, Caregiver, Sedentary behaviour, COM-B

## Abstract

**Background:**

Stroke survivors are highly sedentary; thus, breaking up long uninterrupted bouts of sedentary behaviour could have substantial health benefit. However, there are no intervention strategies specifically aimed at reducing sedentary behaviour tailored for stroke survivors. The purpose of this study was to use co-production approaches to develop an intervention to reduce sedentary behaviour after stroke.

**Methods:**

A series of five co-production workshops with stroke survivors, their caregivers, stroke service staff, exercise professionals, and researchers were conducted in parallel in two-stroke services (England and Scotland). Workshop format was informed by the behaviour change wheel (BCW) framework for developing interventions and incorporated systematic review and empirical evidence. Taking an iterative approach, data from activities and audio recordings were analysed following each workshop and findings used to inform subsequent workshops, to inform both the activities of the next workshop and ongoing intervention development.

**Findings:**

Co-production workshop participants (*n* = 43) included 17 staff, 14 stroke survivors, six caregivers and six researchers. The target behaviour for stroke survivors is to increase standing and moving, and the target behaviour for caregivers and staff is to support and encourage stroke survivors to increase standing and moving. The developed intervention is primarily based on co-produced solutions to barriers to achieving the target behaviour. The developed intervention includes 34 behaviour change techniques. The intervention is to be delivered through stroke services, commencing in the inpatient setting and following through discharge into the community. Participants reported that taking part in intervention development was a positive experience.

**Conclusions:**

To our knowledge, this is the first study that has combined the use of co-production and the BCW to develop an intervention for use in stroke care. In-depth reporting of how a co-production approach was combined with the BCW framework, including the design of bespoke materials for workshop activities, should prove useful to other researchers and practitioners involved in intervention development in stroke.

## Background

Sedentary behaviour is defined as any waking behaviour in a sitting, reclining or lying posture that is characterised by low energy expenditure [1]. High levels of sedentary behaviour are associated with negative health outcomes, including cardiovascular disease mortality [2]. Breaking sedentary behaviour with regular standing increases physical function in frail older adults [3]. UK Chief Medical Officer Physical Activity Guidelines recommend that older adults minimise the amount of time spent being sedentary and break up long periods of inactivity with light physical activity when physically possible or at least standing [4]. Stroke survivors are more sedentary than healthy sex and age-matched controls (10.9 versus 8.2 h/waking day, respectively [5]). Thus, reducing sedentary behaviour has been suggested as a new target for therapeutic intervention after stroke [6].

**Fig. 1:**
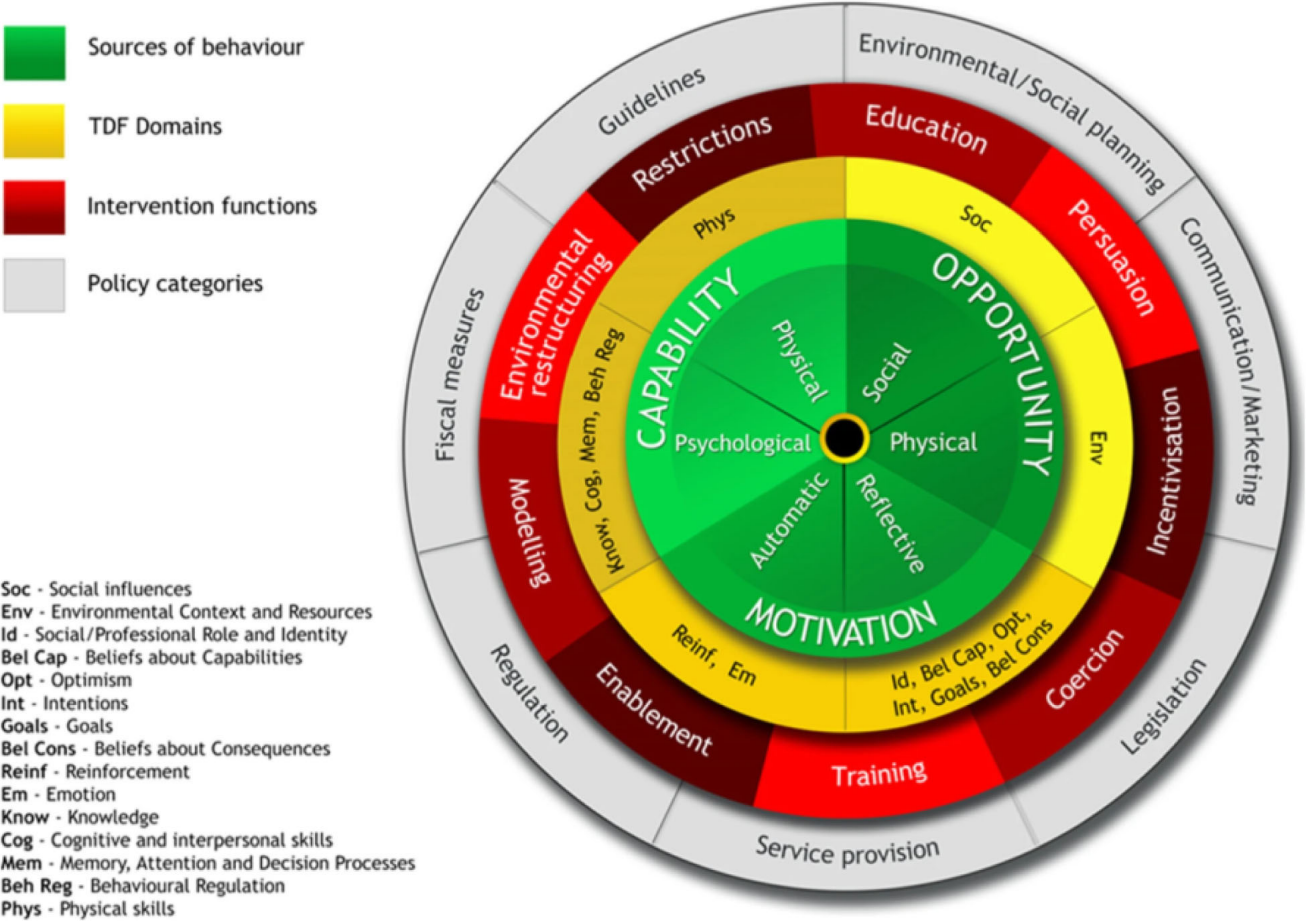
The behaviour change wheel and the theoretical domains framework. Reprinted with permission [9, 19]

The Medical Research Council (MRC) guidance for developing and evaluating interventions outlines the importance of systematically utilising evidence and theory in tandem to develop new interventions [7]. In 2016, an international group of stroke recovery and rehabilitation experts reported that inadequate theoretical intervention development may explain the lack of efficacy of many existing interventions targeting people after stroke [8]. The underutilisation of theory to inform intervention development may be related to the limited guidance available on how to select theory to suit the context [9]. In the context of behavioural theory, the BCW was developed to address this [1]; see Fig. 1.

The capability, opportunity, motivation and behaviour (COM-B) model, which proposes that behaviour is influenced by capability, opportunity, and motivation, forms the central axis of the BCW. The theoretical domains framework (TDF [10];), consisting of 14 psychological domains that influence behaviour, can be used alongside the COM-B model. The COM-B model and TDF can be used to identify barriers to achieving the desired behaviour that an intervention can focus on reducing. The wider BCW then provides a comprehensive guide to producing theoretically informed behaviour change interventions. The BCW framework includes nine intervention functions, seven policy categories, and 93 behaviour change techniques (BCTs) suitable for developing intervention options and content, following the behavioural diagnosis. The framework can be applied across different topics, target groups, and delivery contexts. There are now several examples of the BCW framework being applied to develop interventions, for example, the development of an upper limb exercise intervention in stroke rehabilitation [11] and a workplace sedentary behaviour reduction intervention [12].

Evidence and theory-based intervention development is only one identified approach to developing complex interventions [13]. A recently published taxonomy outlines eight categories; see Table 1. Another of the approaches—partnership—involves active engagement of stakeholders in developing interventions [13]. Taking a partnership approach can facilitate the development of feasible and context-sensitive interventions and may increase the likelihood of developing an intervention that is efficacious [14]. One partnership method is co-production, a process where service users share degrees of power to play an active role in producing or developing goods, services or interventions [15]. Co-production recognises and utilises skills and expertise of various stakeholders, going beyond developing interventions ‘for’ to developing interventions ‘with’ relevant stakeholders including service users and providers [16]. Taking a co-production approach can lead to the development of interventions that focus on changes that are most important to patients, caregivers and staff [17]. This study took a combination approach, by combining partnership with a target population-centred and theory and evidence-based approach. A combination approach has potential to add value beyond what can be achieved by applying either approach independently. The aim of this study was to use co-production together with the BCW framework to develop an intervention to reduce sedentary behaviour after stroke.

**Table 1:** Taxonomy of approaches to developing interventions, adapted from O’Cathain et al. [13]

Category	Definition
1. Partnership	The people for whom the intervention aims to help are involved in decision-making about the intervention throughout the development process, having at least equal decision-making powers with members of the research team
2. Target population centred	Interventions are based on the views and actions of the people who will use the intervention
3. Theory and evidence based	Interventions are based on combining published research evidence and formal theories (e.g. psychological or organisational theories) or theories specific to the intervention
4. Implementation based	Interventions are developed with attention to ensuring the intervention will be used in the real world if effective
5. Efficiency based	Components of an intervention are tested using experimental designs to determine active components and make interventions more efficient
6. Stepped or phased based	Interventions are developed through emphasis on a systematic overview of processes involved in intervention development
7. Intervention-specific	An intervention development approach is constructed for a specific type of intervention
8. Combination	Existing approaches to intervention development are combined

## Methods

### Background to the study and study design

The work reported herein forms part of a programme of research (National Institute for Health Research; RP-PG-0615-20019) to develop and evaluate strategies to reduce sedentary behaviour after stroke and improve outcomes. Findings from three workstreams have fed into the development of an intervention:

- Workstream one involved undertaking a series of systematic reviews to examine views of sedentary behaviour, effectiveness of interventions and effective behaviour change techniques for reducing sedentary behaviour, and the effects of interventions aimed at breaking up sedentary behaviour on health outcomes. Findings from this workstream are reported separately [18].
- Workstream two comprised a qualitative study involving non-participant observations in two-stroke services (inpatient with linked community service), and interviews with stroke survivors at 6 or 9 months post-stroke, their caregivers (if available) and stroke service staff, to explore sedentary behaviours, perceptions of sedentary behaviours, and participants’ capability, opportunity and motivation to address them. Findings from this workstream are reported separately [19].
- Workstream three reported here, utilised co-production principles to develop an intervention, incorporating evidence generated in workstreams one and two, and underpinned by the BCW approach to developing interventions.

The feasibility of the developed prototype intervention has been tested in three UK stroke services (workstream four). A subsequent evaluation for clinical and cost-effectiveness using a multicentre cluster randomised controlled trial (RCT) in 34 UK stroke services with embedded process evaluation (workstream five) will commence in mid-2020. Ethical approval for this study was granted by the Yorkshire & The Humber – Leeds East Research Ethics committee (18/YH/0211) in June 2018.

Co-production principles informing the workshops included: (1) a structured, participatory approach where participants are actively engaged to contribute, (2) a process to ensure all voices were heard and opinions evaluated and acted upon and (3) a process to encourage all participants to contribute to the intervention prototype development. All five workshops lasted 2 h and took place in two UK stroke service settings (West Yorkshire, Edinburgh). Participants included stroke survivors, their caregivers, inpatient and community stroke service staff, and exercise instructors. Throughout the process, the co-production group members appraised the existing evidence, reviewed their experiences and views on sedentary behaviour after stroke, and in collaboration with the research team, contributed to the development of the prototype intervention.

### Participants

The intention was to recruit four-to-six stroke survivors (and their caregivers, if applicable and available) and four-to-six healthcare professionals, public health professionals or volunteers from inpatient and community settings, to participate in all five workshops at the site local to them. Healthcare professionals’ expertise is primarily based on knowledge of the condition, such as outcome, treatment, and stroke care pathways [20]. For stroke survivors and caregivers, knowledge is more often related to the impact and burden of the ongoing impact of stroke on daily life. In addition, all stakeholders were likely to have valuable insights into the potential facilitators and barriers to implementing an intervention to reducing sedentary behaviour after stroke.

Stroke survivors were eligible if they had experienced a stroke within the last 18 months, had currently or previously received treatment or care from a participating stroke service and were able to stand independently or with the assistance of one person. Purposive sampling was used to identify participants, with the aim of including stroke survivors who were diverse in age, gender and mobility status, and staff who varied in discipline, seniority and experience in stroke care, and whether they worked in the inpatient or community setting. A researcher met with all participants prior to the first workshop to provide information about the study, and for them to provide written informed consent.

### Co-production workshop process

Five co-production workshops took place concurrently at both sites from October 2018 to February 2019. Workshops were led by one researcher at each site (JH in West Yorkshire; SM in Edinburgh). At least two other researchers supported facilitation (DJC, CF, RL, LB). The workshops in both sites operated independently of each other, however, they had the same format and content. The workshop schedule permitted flexibility and variation in discussions based on the local context. To ensure consistency between sites, JH and SM attended all workshops across both sites, with the exception of workshop one. See Table 2 for a summary of the content of each co-production workshop.

**Table 2:** A summary of the content of each co-production workshop and post-workshop activity completed by researchers

Focus and content	Links to BCW	Incorporation of evidence	Post-workshop activity
**Workshop 1**			
Introduction to the topic of sedentary behaviour and the intervention target behaviours for each user group	Defining the problem (step 1), identifying the target behaviour (step 2)	Findings from workstream one (health benefits of reducing sedentary behaviour) and workstream two (sedentary behaviour after stroke) incorporated into expert video and infographic	
Introduction to co-production and opportunity to practise a co-production activity—methods for breaking up sedentary behaviour in workshops			Summarising methods and devising a plan for incorporating strategies to reduce and break up sedentary behaviour into subsequent workshops
**Workshop 2**			
Further specification of the target behaviours for each user group in terms of who, where, when, communication etc.	Specifying the target behaviour (step 3)		Summarising the target behaviour (based on BCW Table 3)
Utilisation of ‘personas’ to consider the barriers and facilitators for the three user groups (stroke survivors, caregivers, staff) achieving the target behaviour, via group activity	Identifying what needs to change (step 4)	‘Personas’ developed based on findings from workstream two—related barriers and facilitators to achieving target behaviour	Completing the behavioural diagnosis for each user group (based on BCW Table 4)
**Workshop 3**			
Development and appraisal of solutions to the barriers generated in workshop two, that align with the target behaviour for each user group, via group activity	Identify intervention functions (step 5), identify behaviour change techniques (step 7), identify modes of delivery (step 8)	Infographic illustrating components of effective sedentary behaviour interventions based on findings from workstream one	Coded solutions to intervention functions and delivery methods (based on BCW Table 8) Developed a prototype intervention (specifying how each intervention strategy linked to TDF domain from behavioural diagnosis) based on coded solutions and delivery methods, including application of APEASE^a^
**Workshop 4**			
Appraisal of the proposed intervention, based on the solutions generated in workshop 3, via group activity and individual validation activity	Identify intervention functions (step 5), identify behaviour change techniques (step 7), identify modes of delivery (step 8)		Calculated scores from validation activity and summarised data Revised prototype intervention based on workshop data Commenced BCT coding of the prototype intervention Developed a selection of prototype materials
**Workshop 5**			
Review of prototype materials via group activity	Identify modes of delivery (step 8)		Summarised appraisal of prototype materials and revised prototype intervention in line with this BCT coding of prototype intervention operationalised the intervention—development of all materials
Reflection on participation in co-production workshops			Narratively summarised reflections

^a^APEASE, criteria for appraising intervention options—affordability, practicability, effectiveness and cost-effectiveness, acceptability, safety and equity

Workshop one was conducted separately for staff, and for stroke survivors and their caregivers. This was to introduce the concepts of sedentary behaviour and co-production in a more comfortable environment, and to encourage both groups to think about ways of working collaboratively, on an equal basis, as opposed to a staff or expert-led approach to patient and public involvement, which may be more familiar to some participants. In this workshop, participants were actively involved in co-designing and then utilising (in each subsequent workshop) progressive activities involving standing and moving during the workshops. This increased practical understanding of the simple approaches that could be used in everyday life as well as engaged the participants in problem-solving and decision-making, ahead of co-producing the intervention. Each subsequent workshop was conducted with both groups and included three ‘elements’: (1) evidence .and information provision, (2) utilising knowledge and experience of group members via bespoke workshop activities informed by the BCW process, and (3) evaluating the process.

#### Strategies for communicating evidence

Key findings from workstream 1 and workstream 2 of the wider project were fed into the workshops at appropriate points to allow the intervention development to be informed by up-to-date, relevant evidence. For example, case studies, referred to during the process as ‘personas’ were developed as a method of communicating findings from workstream two about the barriers and facilitators of reducing sedentary behaviour (stroke survivor personas) and supporting stroke survivors to reduce sedentary behaviour (staff and caregiver personas). Infographics were used to communicate findings from systematic reviews conducted in workstream one, related to components of effective interventions, in a user-friendly and engaging format. Examples of these personas and infographics can be found in Additional file 1. Verbal and written summaries of discussions and updates on developments since the earlier workshops were also provided, and often utilised within subsequent workshop activities. Examples of written summaries of discussions focused barriers to achieving the stroke survivor target behaviour are in Additional file 2.

#### Utilising knowledge and experience of group members during workshop activities

Workshop activities were informed, to varying degrees, by BCW worksheets [21]. For example, workshop two activities generated insights for specifying the target behaviours and to complete a behavioural diagnosis for each of the groups. Activities in later workshops focused on open and creative idea generation. Whilst these activities were less directly aligned to the BCW the data generated was applied to the relevant BCW steps, outside of the workshops (see the analysis section). Although the BCW was used to inform the workshop tasks and analyse workshop data, technical language was avoided by facilitators and in workshop materials, thus, it may not have been obvious to the participants that the process was driven by theory. Bespoke materials were developed for each activity. Selected examples of workshop materials are provided in Additional file 3.

At each workshop, participants worked in smaller pre-allocated groups, each with a facilitator. The facilitators supported the involvement of all participants in discussions, e.g., those with hearing difficulties. Facilitators utilised topic guides based on evidence from earlier workstreams during discussions; see Additional file 4. Smaller groups fed back key discussion points to the wider group, to share ideas and to foster a sense of ownership of the developing intervention across all participants.

#### Evaluating the process

A range of methods were adopted to evaluate involvement in the workshop process, including questions and feedback, researcher reflections, a participant group discussion during the final workshop, and evaluation forms. Feedback from the evaluation forms was applied to improve participants’ experience in subsequent workshops. All participants were given a certificate of attendance following the final workshop to recognise their input and achievement.

### Data collection and analysis

All completed worksheets and forms described in the “Co-production workshop process” section, and workshop discussions, were utilised as data. Additionally, each small group discussion was audio-recorded and transcribed by one of three researchers (JH, SM, JFH). Data collection, analysis and intervention development were iterative: following each workshop, data were analysed and interpreted ahead of the next workshop, and used to inform both the content of subsequent workshops and intervention development. Data from workshops two and three were coded using NVivo 11 by three researchers (JH, SM, JFH) and discrepancies were discussed with a fourth researcher (RL); a deductive approach to analysis was taken which involved data being coded according to pre-defined categories/theories/techniques as part of the BCW process. Further detail on how the data from each workshop were analysed, and how this informed ongoing intervention development, is included in the ‘post workshop activity’ column of Table 2.

## Results

### Participant characteristics and workshop attendance

Forty-three people participated in the co-production workshops including 17 staff, 14 stroke survivors, six caregivers, and six researchers (workshop facilitators; JH, SM, CF, DJC, RL, LB). At least three researchers were present at each workshop to facilitate the small-group discussions and activities. See Table 3 for an overview of attendance at each workshop in each location.

**Table 3:** Co-production workshop attendance in West Yorkshire and Edinburgh

		West Yorkshire	Edinburgh
Workshop 1—*October 2018*	Stroke survivor	5	4
	Caregiver	3	1
	Staff	6	7
Workshop 2—*November 2018*	Stroke survivor	5	6
	Caregiver	2	3
	Staff	5	3
Workshop 3—*December 2018*	Stroke survivor	6	7
	Caregiver	2	2
	Staff	5	6
Workshop 4—*January 2019*	Stroke survivor	4	6
	Caregiver	3	2
	Staff	5	6
Workshop 5—*February 2019*	Stroke survivor	6	6
	Caregiver	3	2
	Staff	5	5

At the time of the first workshop, the average age of the stroke survivor participants was 72 years (range of 56 to 83 years) and the average time since the event of their stroke was 10 months (range of 4 to 15 months). The average age of caregivers was 68 years (range of 54 to 83 years). Other stroke survivors and caregiver participant characteristics are detailed in Table 4. Staff participants included physiotherapists, therapy assistants, occupational therapists, registered nurses, healthcare support workers, exercise instructors and volunteers. Aside from the exercise instructors, all staff worked at an inpatient stroke unit or for a linked community stroke service, and varied in seniority. Ten staff participants had more than 5 years’ experience in stroke care and the majority (15) were female.

**Table 4:** Stroke survivor and caregiver participant characteristics

	Number (percentage)	
	Stroke survivors	Caregivers
Female	6 (43%)	4 (67%)
Presence of aphasia	3 (21%)	
Capability to stand independently	13 (93%)	
Retired	11 (79%)	4 (67%)
Full-time employed	2 (14%)	1 (17%)
Unemployed	1 (7%)	0 (0%)
Stroke survivors’ spouse		5 (83%)
Stroke survivors’ daughter		1 (17%)

### Feedback on the co-production process

Overall satisfaction with the workshops (1 = not at all satisfied, and 5 = very satisfied), averaged across workshops, sites and participants, was 4.8. Overall satisfaction with the strategies used to encourage standing and moving during the workshops was 4.7. The frequency of words selected to best represent experience of the workshops are illustrated in Table 5.

**Table 5:** Responses to request to ‘circle at least 3 words that best represent your overall experience of today [the co-production workshop]’. Responses are collated across both sites and all five workshops

Frequency of word selection	
40+	Interesting, thought-provoking, useful
30-39	Valuable, realistic
20-29	Enjoyable, inspiring
10-19	Challenging, rushed, clear
1-9	Difficult, fascinating, exciting, new, fun, entertaining, empowering, stimulating, overwhelming, too short, too structured, exhausting, vague, intimidating
0	Boring, confusing, too long, unfocused, overambitious, waste of time

Positive feedback about participating in the co-production workshops included feeling that the contributed views, experience and expertise were valued in the intervention development process, ‘demystifying research’ and gaining an insight into the processing of developing interventions and being given an opportunity to interact and share experiences with other people who have had a stroke. Participants also reported that being part of the process had a positive influence on their motivation and capability to (support stroke survivors to) reduce sedentary behaviour. Negative feedback included a perception that workshop participants were all capable of—and motivated to—reduce sedentary behaviour (stroke survivors) and support stroke survivors to reduce sedentary behaviour (caregivers and staff).

### Intervention development: increasing standing and moving after stroke

**Fig. 2:**
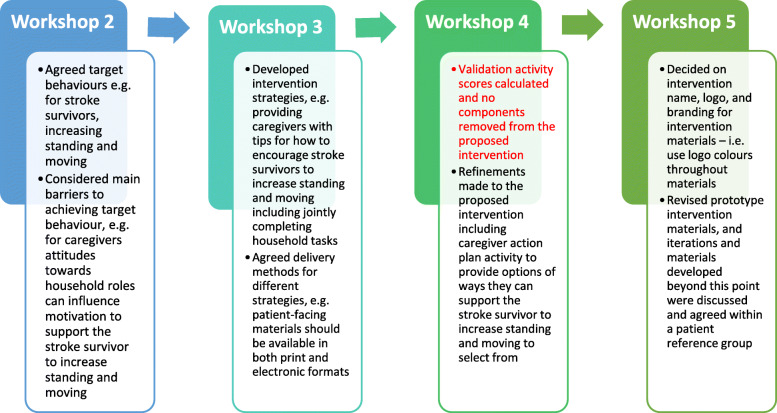
Examples of how data from each workshop contributed to intervention development

This section describes the process of intervention development. The BCW steps are presented sequentially for clarity however in reality the activities fluid and non-linear. Co-produced intervention strategies were coded to the BCTs and intervention functions, rather than being used to structure the workshop tasks. Figure 2 outlines the key outputs from each co-production workshop in relation to intervention development.

#### BCW stage 1: understanding of the behaviour

Researchers’ work to understand the behaviour took place prior to the workshops, and during workshops one and two. Data from workshop two was analysed to specify the target behaviour and complete behavioural analyses for the three target behaviours.

##### Step 1: defining the problem

The intervention aims to address the ‘problem’ of high levels of sedentary behaviour in people after stroke. This was defined by the research team as part of the process of acquiring funding for the research and was communicated to the participants involved in the workshops as part of recruitment for the workshops and reiterated during workshop one.

##### Step 2: identifying the target behaviour

Target behaviours were identified for each user group: to increase standing and moving (stroke survivors), and to support stroke survivors to increase standing and moving (staff and caregivers). Whilst the target behaviour was identified prior to the co-production work, participants’ preferred terminology was discussed and agreed during the second co-production workshop. There was a consensus to avoid the word ‘sedentary’ in the target behaviours and in intervention materials targeted at stroke survivors and caregivers due to a perception that the word is complex, technical, ambiguous and can have negative connotations, for example, ‘lazy’. Prior to engaging in the workshops some participants misunderstood sedentary behaviour and associated this with a lack of physical, cognitive or social activity. Participants agreed that ‘increase standing and moving’ is simple and understandable, and reframes the issue in a positive way, i.e. it is a goal to work towards.

##### Step 3: specifying the target behaviour

During the second co-production workshop, participants discussed the target behaviour for each user group of the intervention, including considering where and when the target behaviour should be performed. Following the workshop in each location, data from the discussion was coded and summarised by the research team, to inform the specification of each target behaviour. As an example, the stroke survivor target behaviour is aimed at all stroke survivors admitted to an inpatient stroke unit who are safe and able to stand either independently or with the assistance of one person. Stroke survivors should aim to stand and move at regular intervals throughout the day however this will be tailored to individual stroke survivors’ capability, safety, and circumstances.

##### Step 4: identifying what needs to change

A ‘behavioural diagnosis’ activity was completed during workshop two, which involved identifying barriers and facilitators to achieving the target behaviour for two different stroke survivor, caregiver and staff member ‘personas’, which were developed based on the evidence and insight generated during earlier work streams. Following the workshop, two researchers coded the data from this activity into capability, opportunity, motivation and the TDF domain categories. The behavioural diagnosis facilitated the identification of which COM-B and TDF domains the intervention should target for each user group, based on the absence or presence of barriers pertaining to each domain; see Table 6.

**Table 6:** TDF domains that are targeted within the ‘Get Set, Go’ intervention

TDF domain	Targeted	Targeting which user groups
Physical skills	Yes	Staff, caregivers
Knowledge	Yes	Stroke survivors, staff, caregivers
Cognitive and interpersonal skills	Yes	Stroke survivors, staff caregivers
Memory, attention and decision processes	Yes	Stroke survivors, staff
Behavioural regulation	Yes	Stroke survivors, staff, caregivers
Environmental context and resources	Yes	Stroke survivors, staff, caregivers
Social influences	Yes	Stroke survivors, caregivers
Professional/social role and identity	Yes	Stroke survivors, staff, caregivers
Beliefs about capabilities	Yes	Stroke survivors, staff, caregivers
Optimism	No	
Beliefs about consequences	Yes	Stroke survivors, staff, caregivers
Intentions	Yes	Stroke survivors
Goals	Yes	Stroke survivors, staff, caregivers
Reinforcement	Yes	Caregivers
Emotions	Yes	Stroke survivors, staff, caregivers

As an example, barriers to caregivers achieving the target behaviour across the COM-B domains included a limited understanding of how to support stroke survivors to increase standing and moving (capability), caregivers having other responsibilities which limit the time they have available to support the stroke survivor (opportunity), and a perception that there are risks to supporting the stroke survivor to increase standing and movement, such as an increased falls risk, that outweigh the potential benefits (motivation). An example of a completed behavioural diagnosis is presented in Additional file 5. The research team developed summaries of the main barriers to achieving the target behaviour for each user group; see Additional file 2 for the stroke survivor example.

#### BCW stage 2: intervention options

During workshop three, participants utilised the summaries of the main barriers to achieving the target behaviour to identify, and then appraise, ‘solutions’ to the barriers, including delivery methods. Following the workshop, the research team reviewed the data and applied the APEASE criteria to co-produced intervention components to develop a prototype intervention. All intervention components were based on solutions and delivery methods favoured during the workshops and were ‘coded’ to TDF domains to ensure that the prototype intervention addressed the domains identified as being important to target following the behavioural diagnosis (workshop two). During the workshop, four participants appraised the draft intervention components via a validation activity. Prior to the workshop, the researchers agreed that any proposed intervention component that had more ‘no’ than ‘yes’ responses across all participants would be removed from the proposed intervention; no intervention components were removed as a result of this appraisal activity. The proposed intervention was iteratively refined based on a review of workshop four and five qualitative data. The developed intervention was then retrospectively coded for intervention functions and policy categories.

##### Step 5: identify intervention functions

The intervention strategies co-produced in workshop three and refined in workshop four were subsequently coded to five of the nine intervention functions included within the BCW [21]. The ‘Get Set, Go’ intervention functions are detailed in Table 7 with examples of intervention strategies that align with the function.

**Table 7:** ‘Get Set, Go’ intervention functions

Intervention function	Definition	Example intervention strategy
Education	Increasing knowledge or understanding	Providing information to staff, stroke survivors and caregivers about the benefits of standing and moving
Persuasion	Using communication to induce positive or negative feelings or stimulate action	Deliver messages via authoritative source
Training	Imparting skills	Upskilling staff in how to support stroke survivors to increase standing and moving
Environmental changes	Changing the physical or social context	Suggestions provided with regards to how to adapt the home environment
Enablement	Increasing means/reducing barriers to increase capability or opportunity	Senior colleagues being supportive of delivering the intervention

##### Step 6: identify policy categories

The intervention is intended to be delivered at a service level, and thus incorporates the policy category ‘guidelines’ as this involves creating documents that recommend or mandate practise including all changes to service provision. None of the other BCW policy categories were applicable to the developed intervention.

#### BCW stage 3: content and implementation options

As with the BCW intervention functions and policy categories (stage two), the co-produced intervention based on the behavioural diagnosis was retrospectively coded for behaviour change techniques and delivery modes.

##### Step 7: identify behaviour change techniques

Following the co-production of the intervention components, three researchers (JH, SM, JFH) collaboratively coded BCTs evident within each intervention component. For example, caregivers completing an action planning activity which involves considering challenges to achieving the target behaviour and how to overcome them was coded as problem solving. Other intervention components were further specified to include additional BCTs, for example, information about others’ approval (BCT 6.3) was applied to staff training. The intervention incorporates 34 BCTs from the behaviour change technique taxonomy v1 [22]. The included BCTs were from 11 of the 16 categories: goals and planning, feedback and monitoring, social support, shaping knowledge, natural consequences, comparison of behaviour, associations, repetition and substitution, comparison of outcomes, antecedents, identity and self-belief. No BCTs were included from the categories: reward and threat, regulation, scheduled consequences and covert learning. See Table 8 for some examples of BCTs across a range of categories, including which TDF domains they target and how they are operationalised in the intervention.

**Table 8:** Selected BCTs and examples of how operationalised in the intervention

Example BCT	TDF domains	Example operationalisation
1.2 Problem solving (goals and planning)	Skills, intentions, goals, behavioural regulation	Caregivers consider challenges to achieving target behaviour in ‘action planning’ activity
2.3 Self-monitoring of behaviour (feedback and monitoring)	Intentions, goals, behavioural regulation	Monitoring sheets provided for patients to record standing and moving activity
3.2 Social support—practical (social support)	Social influences	Providing examples of how caregivers can provide practical help to stroke survivors
4.1 Instruction on how to perform a behaviour (shaping knowledge)	Knowledge, skills, memory/attention/decision-making processes	Advise staff on how to deliver intervention components during training session
5.1 Information about health consequences (natural consequences)	Knowledge, beliefs about consequences	Inform staff and stroke survivors about the health benefits of standing and moving after stroke
6.3 Information about others’ approval (comparison of behaviour)	Social / professional identity and role, beliefs about capabilities, beliefs about consequences	Informing staff that senior colleagues approve of supporting patient to increase standing and moving
8.7 Graded tasks (repetition and substitution)	Behavioural regulation	Increasing stroke survivors’ standing and moving target over time, dependent on ability
9.1 Credible source (comparison of outcomes)	Social/professional role and identity, beliefs about consequences	Advice relating to standing and moving provided to patients and caregivers by professionals
15.1 Verbal persuasion about capability (self-belief)	Beliefs about capabilities, behavioural regulation	Informing stroke survivors of their ability to stand and move

##### Step 8: identify mode of delivery

Modes of delivery were initially discussed during workshop three and iteratively refined alongside the intervention components based on data from workshops four and five. Most of the intervention is delivered face-to-face: some takes place at a group level (e.g. staff training) whereas other components are delivered at an individual level. The intervention also includes written materials, which are also available online.

## Discussion

This paper reports on an extensive and robust process to develop a comprehensive intervention strategy to target sedentary behaviour after stroke. Intervention studies targeting sedentary behaviour after stroke to date are few in number and report inconclusive findings. Novel intervention approaches are urgently needed. Using a combined co-production and BCW approach, this study has made a substantial and formative contribution to the literature in the field. In line with MRC guidance, the intervention was tested and refined in a feasibility study and will be evaluated for effectiveness in a multi-site cluster randomised controlled trial [7].

In contrast to some studies reporting challenges in retaining patients and carers in co-production work (e.g. [23]), this study reported a high retention of participants across the workshops. The research team invested time to build trust and positive relationships with the participants, and sought to foster a sense of ownership of the intervention development amongst the participants, by emphasising their expertise and ‘power’ to meaningfully contribute. Feelings of ownership and a perceived ability to contribute are recognised as important factors influencing engagement in and success of co-production approaches [14]. The participants in the present study indicated that being part of the workshop process had been a positive experience. This is consistent with other co-production and co-design projects [24]. Peer support groups have been identified by stroke survivors as facilitating adjustment to life after stroke by, for example, providing an opportunity to learn from other stroke survivors about coping with the impacts of stroke [25]. Increased peer support was an unintended but positive outcome of the co-production work, beyond the primary aim of developing an intervention to reduce sedentary behaviour after stroke.

The co-production approach utilised during this study was guided by the BCW framework for developing interventions. Utilising the BCW was highly beneficial as it has resulted in an intervention that can be described using defined terminology (e.g. BCTs) which increases intervention replicability and will inform evaluation [21]. The BCW also provided structure to the co-production workshops and related intervention development. Whilst co-production is founded on various principles such as greater equality in the relations between users and professionals [26], there are no set procedures or methods for using a co-production approach to develop an intervention. The BCW approach helped to develop ‘milestones’ for each workshop and ensured defined progress was achieved following each workshop.

Conducting the behavioural diagnosis (stage one of the BCW) worked particularly well as a group co-production activity, utilising evidence from earlier work streams through the ‘personas’ and drawing on the participants’ experience and expertise to prioritise the key barriers and facilitators to achieving the target behaviours. However, the behavioural diagnosis resulted in barriers across almost all domains of COM-B and TDF, meaning that all intervention functions and policy categories listed in the BCW framework were potentially relevant. The behavioural diagnosis is intended to narrow down intervention options by focusing on one or two TDF domains such as knowledge and emotions [21], but the complexity of the behaviours meant that barriers permeated across all domains. Other researchers who have developed interventions in a stroke care setting and/or focused on activity using the BCW framework have also reported this issue (e.g. [11, 27]).

There was a degree of tension between stage two and three of the BCW process and the co-production approach. It was felt that working directly with intervention functions and BCTs within the workshops might be restrictive and stunt creativity. To counteract this, a bottom-up approach to solution generation was undertaken, based on the behavioural analysis, and the intervention was retrospectively coded into BCW intervention functions, BCTs, policy categories and delivery methods. The authors of the BCW framework recognise the non-linearity of intervention development and recommend a flexible application [21]. Only one policy category—guidelines—was selected in the present study. This was partly due to being limited by the evaluation design for the subsequent cluster RCT; it was not deemed appropriate to include policy changes that are delivered wider than service level, as this would risk contamination across the control sites during the RCT. Additionally, it has been suggested that the inclusion of policy categories is not essential to an intervention, and that policy changes might be more appropriately considered as an intervention function option within the BCW rather than as a discrete step in the process [28].

Co-production approaches and the BCW framework are both resource-intensive methods. Across all participants, the workshops consisted of over 250 h of participant time. Additionally, three researchers (JH, SM, JFH) spent invested significant time and effort preparing materials for the workshops and analysing data over a 6-month period. For example, BCT coding took three staff 26 h each. It is felt that the time and resources invested have led to the development of a comprehensive intervention that exceeds the needs of the intended users; however, this investment would not have been possible without the funding received to develop and test the intervention.

### Strengths and limitations of this work

We acknowledge some limitations of the intervention development process. Workshop participants were not necessarily representative of the general stroke survivor population that the intervention is designed for. The participants were a highly motivated group and consequently, the intervention strategies will appear more acceptable to them. A recognised and common concern relating to co-production is that such processes may reinforce inequalities as more educated citizens are more likely to be receptive to engaging due to perceptions of their capability to contribute [29]. Work is required to understand how best to engage more disadvantaged individuals within co-production work. Additionally, stroke survivor participants were, on average, 10 months post-stroke at the time of the first workshop, whereas the intervention will target stroke survivors in the immediate stages post-stroke (in the inpatient setting) and for 12 weeks post-hospital discharge.

A limitation of this paper is that the intervention is not described in complete detail. There are plans to evaluate effectiveness in a cluster randomised controlled trial and therefore to avoid contamination across control sites, we have avoided disclosing detailed information about the various components of the intervention. To date, there is limited evidence about the clinical effectiveness of co-produced interventions [14, 23]. An ongoing, robustly designed study aims to generate evidence related to measures of co-production processes and their outcomes through comparative case studies of nine co-production projects [30]. Future research could examine this further by evaluating co-produced interventions for effectiveness.

The main strength of this study is the in-depth reporting of how a co-production approach was combined with the BCW framework to develop an intervention. Our methods and bespoke materials may help other researchers and intervention developers seeking to combine co-production and the BCW to intervention development. Researchers need to publish detailed reports of how they have combined co-production with the BCW or other theoretical frameworks, to advance the field of complex intervention development. Given the varied benefits that both co-production and the BCW can offer, it is plausible that combining these approaches might result in more feasible, acceptable and ultimately effective interventions than utilising either approach in isolation. We hope to offer some insight into this following our planned cluster randomised controlled trial.

## Conclusions

This paper reports on an intervention development process combining co-production and the BCW. The study found that, although there were challenges, combining co-production and the BCW is feasible and has multiple benefits including the BCW providing a structure to the co-production process, and the developed intervention viewed as being feasible to deliver through stroke services by participants. This study provides practical examples of how to use the BCW to guide the co-production of an intervention, including the design and utilisation of bespoke materials. The intervention has been tested and refined in a feasibility study, and will be evaluated for effectiveness in a multi-site cluster RCT.

## Supplementary information

**Additional file 1.** Example personas and infographics. These are examples of materials developed to communicate findings from previous work streams.

**Additional file 2.** Example materials specifying target behaviour and barriers to achieving the target behaviour. These are written summaries of discussions and updates on developments since the earlier workshops.

**Additional file 3.** Selected examples of workshop materials.

**Additional file 4.** Topic guides. These are prompts for workshop facilitators based on evidence from earlier work streams.

**Additional file 5.** Example of completed behavioural diagnosis.

## Data Availability

The dataset generated and analysed during the current study are not publicly available to preserve the anonymity of research participants.

## Abbreviations

BCTs: Behaviour change techniques
BCW: Behaviour change wheel
COM-B: Capability, opportunity, motivation-behaviour
RCT: Randomised controlled trial
TDF: Theoretical domains framework
