# Supplementary figures and images for: A co-production approach guided by the behaviour change wheel to develop an intervention for reducing sedentary behaviour after stroke
Source: Pilot Feasibility Stud. 2020 Aug 17;6:115. doi: 10.1186/s40814-020-00667-1 (PMC7429798; doi:10.1186/s40814-020-00667-1)

Examples of personas and infographics – communicating evidence from earlier workstreams


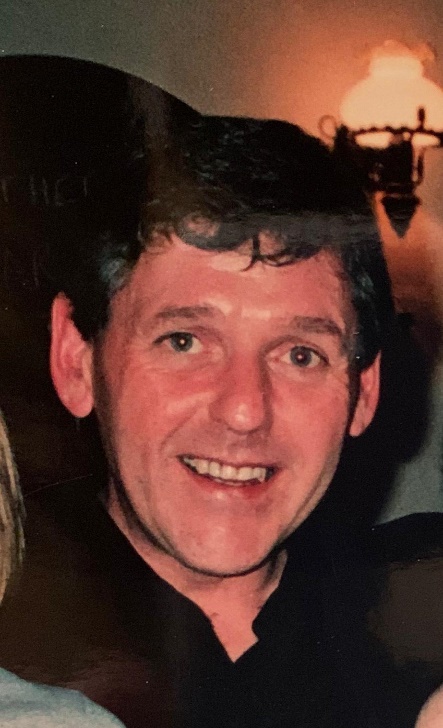

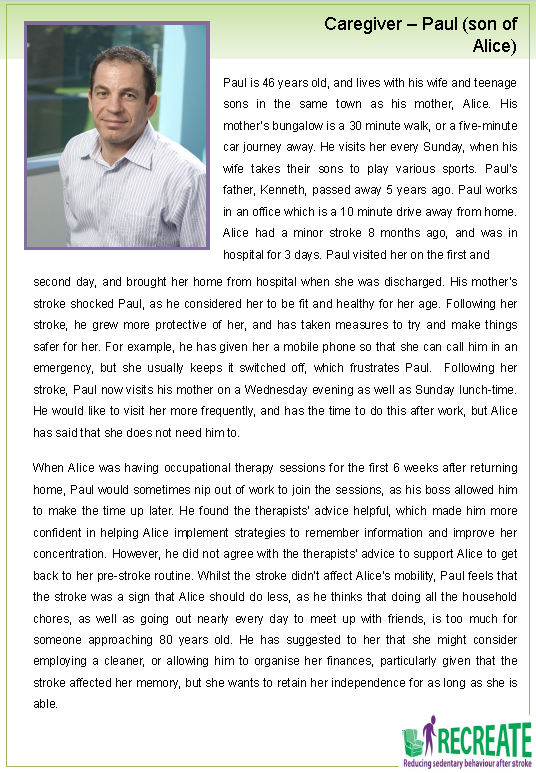


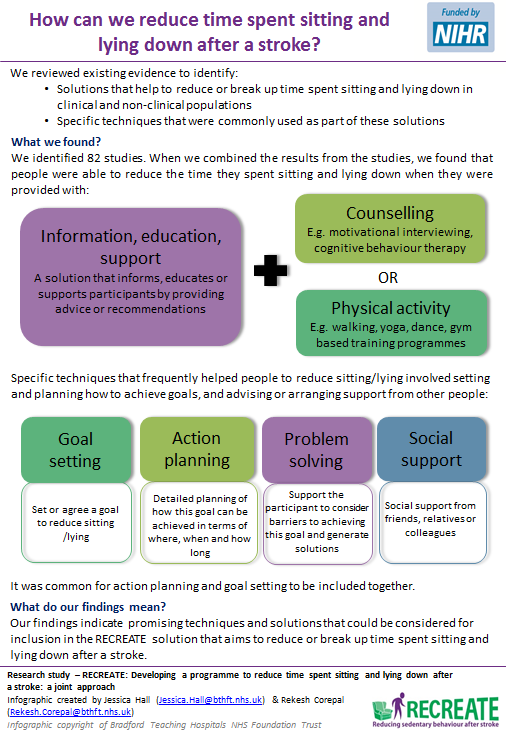

Supplement: Supplementary file 1 — Additional file 1. Example personas and infographics. These are examples of materials developed to communicate findings from previous work streams. [file 40814_2020_667_MOESM1_ESM.docx]

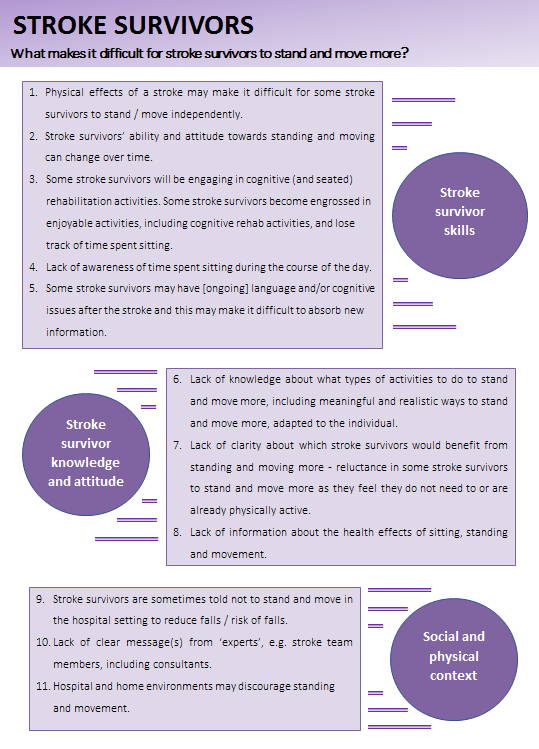

Supplement: Supplementary file 2 — Additional file 2. Example materials specifying target behaviour and barriers to achieving the target behaviour. These are written summaries of discussions and updates on developments since the earlier workshops. [file 40814_2020_667_MOESM2_ESM.docx]
