# Supplementary material for: A co-production approach guided by the behaviour change wheel to develop an intervention for reducing sedentary behaviour after stroke
Source: Pilot Feasibility Stud. 2020 Aug 17;6:115. doi: 10.1186/s40814-020-00667-1 (PMC7429798; doi:10.1186/s40814-020-00667-1)

Worksheet for barriers and facilitators task (caregiver group) – workshop 1


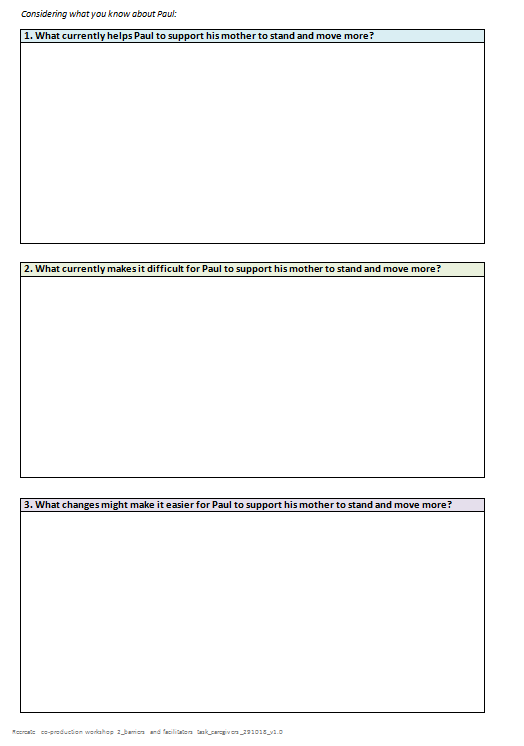


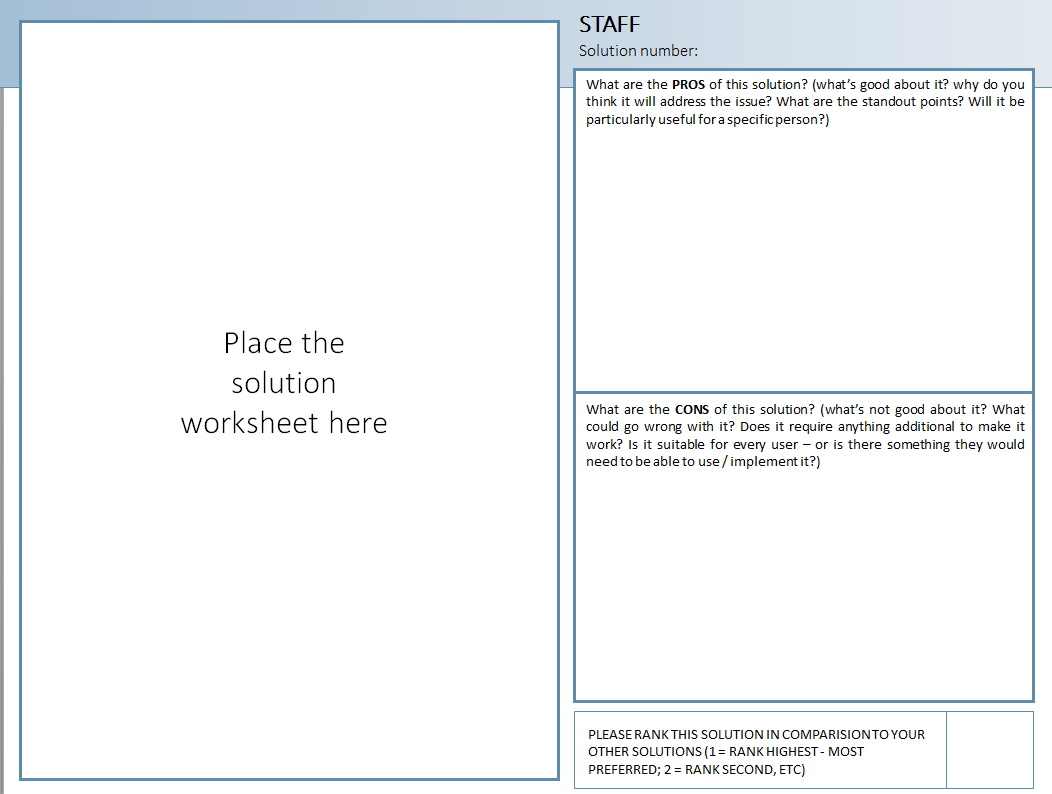

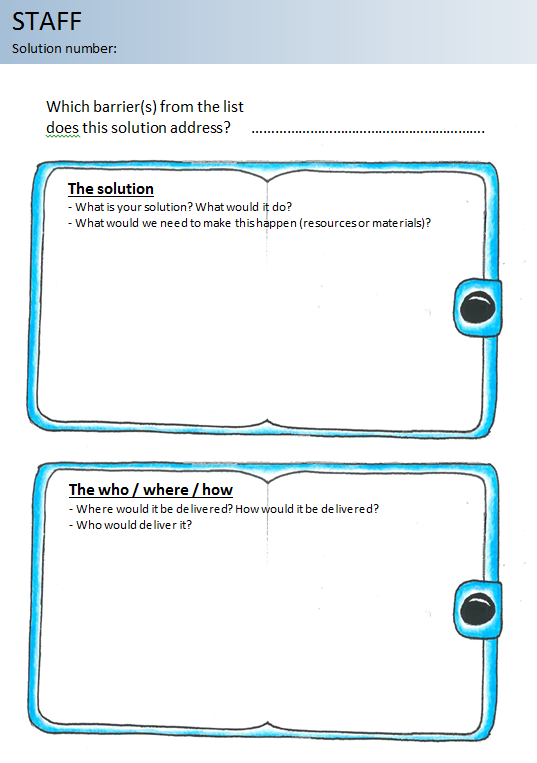
Worksheets for generating for appraising solutions activities (staff) – workshop 3

An extract from individually-completed validation activity – workshop 4

| 1. Education about the importance of standing and moving after stroke  Bespoke to staff, stroke survivors, and family and friends, including: | | YES | NO |
| --- | --- | --- | --- |
| COMPONENTS | | | |
| 1a | Health consequences of sitting, standing and moving |  |  |
| 1b | How to stand and move safely including relationship to falls risk |  |  |
| 1c | Considering frequency of and suggestions for standing and moving |  |  |
| 1d | Staff specific content about how to integrate the solution(s) into routine practice |  |  |

Appraising prototype intervention material example – workshop 5


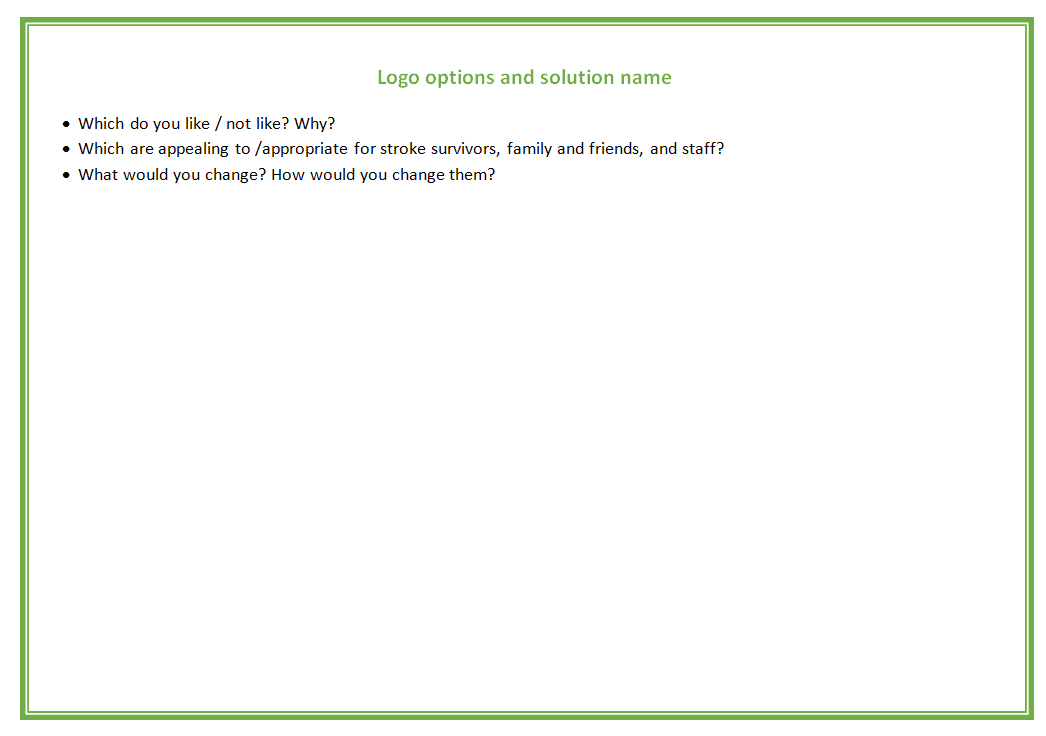

Supplement: Supplementary file 3 — Additional file 3. Selected examples of workshop materials. [file 40814_2020_667_MOESM3_ESM.docx]
