# Supplementary material for: A co-production approach guided by the behaviour change wheel to develop an intervention for reducing sedentary behaviour after stroke
Source: Pilot Feasibility Stud. 2020 Aug 17;6:115. doi: 10.1186/s40814-020-00667-1 (PMC7429798; doi:10.1186/s40814-020-00667-1)
Supplement: Supplementary file 4 — Additional file 4. Topic guides. These are prompts for workshop facilitators based on evidence from earlier work streams. [file 40814_2020_667_MOESM4_ESM.docx]

Topic guide – Stroke survivors

| Barrier 1 – (perceived) capability | - Therapy staff know what stage patients are at in their recovery / best placed to assess when suitable to receive targeted intervention? - Fatigue appears to be a factor in reducing patient ability to break up sitting time, but for many people this does appear to reduce over time. How to accommodate this? - Reduced confidence and anxiety experienced after stroke re standing movement (partly due to safety concerns) – overcome via pacing? Grading? - Seasonality influences perceived capability - fear of falling on slippery external surfaces, reduced day light hours. |
| --- | --- |
| Barrier 2 – capability and attitude over time | - Therapy provision is often adapted to meet the needs of individual patients. How to capitalise on this? - The intervention might need to emphasise the importance of continuing to work on mobility to increase the likelihood that patients are capable of standing and moving. |
| Barrier 3 – importance of sitting | - How to balance intervention with rest / fatigue management. Therapy staff encourage ‘pacing’ of activities which fits with the SB agenda. |
| Barrier 4 – awareness of sitting | - Similarities in daily patterns of sitting and movement across patients (i.e. more activity in the morning, slowing toward the end of the day. Focus on ‘problem’ times of day / encourage throughout the day? - There are some strategies to monitor movement (e.g. diaries, goal setting, FitBit) – but not sedentary time. Could this existing practice be tapped into? |
| Barrier 5 | - |
| Barrier 6 – knowledge about what to do | - Inpatient – potential for staff / caregivers to take patients off the ward? - Activity suggestions need to be regular and often e.g. sit-to-stands, making cup of tea, etc. rather than going for a walk a few times a week or attending an exercise class. Whilst these are beneficial they are unlikely to make a dent in total SB. |
| Barrier 7 – need | - Some stroke survivors make a good recovery, and return to pre-stroke activity, and don’t feel they are in need of the intervention. |
| Barrier 8 - health | - Patients tend toward wanting to regain ‘normality’ and independence, and this is highlighted as a key recovery objective / goal – how to capitalise on this? - How can we engage those patients who revert to a negative post-stroke lifestyle (negative habitual behaviour) and have no interest in spending less time sitting? |
| Barrier 9 | - |
| Barrier 10 – lack of message from experts | - How can patients be made aware that they have permission to move (relates to   ward culture)? How can this be managed safely?   - Patients engage in a range of therapy activities in different areas around the ward (time limited). There does not appear to be any activity ‘prescribed’ to take place outside of formal therapy sessions. - Staff engage in routine activity that encourages patients to break up sitting time, but breaking sitting is not a motivator / objective of these activities and thus this is not communicated to patients (e.g. encouraging independent bathroom use). - There are existing opportunities that staff talk about lifestyle behaviours (e.g. discharge visit from the community) – discuss potential for discussion to occur at these time-points. - Some therapy provision is delivered in a way that makes it a sedentary activity. |
| Barrier 11 – env (hosp and home) | - Patients spend a large proportion of their day at the bedside lying or sitting down – could this space be utilised as a place for the intervention to take place. What movement-related activities would it be possible to offer patients? - Some patients are attached to equipment while they are in the inpatient setting, however are capable of moving – how to involve these patients? - When patients return home there appears to be more opportunity to move around. However home environments of patients vary greatly. - Visitors tend to be more likely to be present on the ward / inpatient setting in the evening and at weekends. These times are generally outside of times when therapy staff are present on the ward. |
| Barrier 12 –social support | - Those patients who tend to do more (generally – e.g. going for lunch, participating in exercises classes) tend to have access to a social network (which they value and enjoy). Not all patients will have access to this. However, it does appear to be an asset – motivator particularly. |

Activity 2

Consider for each solution, where appropriate:

- **Affordability and cost-effectiveness:** How much might this solution cost to develop and implement? Do you think it’s worth the money, or could it be better spent?
- **Practicability:** How easy difficult will it be to implement in practice? What issues might there be? Will it fit within the context of stroke survivors’ daily lives? Can it be adapted to fit different stroke service contexts?
- **Effectiveness:** How much of an impact do you think it will have on encouraging stroke survivors to stand and move more? Is it likely to lead to a small or a bigger change?
- **Acceptability:** How might stroke survivors react to it? Do you think they’ll like it? Might they have any negative reactions to it?
- **Safety / side effects:** Might there be any safety concerns? Might there be any unintended consequences or side effects?
- **Equity**: Might it work better / have more impact / be preferred by some stroke survivors rather than others? Is it possible for the solution to be accessible by all stroke survivors we are targeting the programme at?
