# Supplementary material for: A co-production approach guided by the behaviour change wheel to develop an intervention for reducing sedentary behaviour after stroke
Source: Pilot Feasibility Stud. 2020 Aug 17;6:115. doi: 10.1186/s40814-020-00667-1 (PMC7429798; doi:10.1186/s40814-020-00667-1)
Supplement: Supplementary file 5 — Additional file 5. Example of completed behavioural diagnosis. [file 40814_2020_667_MOESM5_ESM.docx]

| **COM-B** | **TDF** | **Facilitators** | **Barriers, and what needs to happen to achieve the desired behaviour** | **Relevance of domain / need for change?** |
| --- | --- | --- | --- | --- |
| Physical capability | Physical skills | Skilled in physically supporting movement as receive training and support from stroke service staff | Additional training and support from staff required, including demonstration, to enhance physical skills in supporting stroke survivor to stand and move more in a safe way. There is a particular need for caregivers who don’t currently receive any support from staff (due to their circumstances, or stroke survivor not requiring (physio)therapy | Yes / relevant |
| Psychological capability | Knowledge | Some caregivers are knowledgeable regarding potential strategies for supporting stroke survivors to break up sitting time | Not all caregivers are knowledgeable – require education relating to benefits of standing, and strategies / suggestions for supporting stroke survivors to increase standing  Need education for caregiver as well as stroke survivor to empower caregivers to also change their own (lifestyle / sedentary) behaviour | Yes / relevant |
|  | Cognitive and interpersonal skills | Some caregivers are able to identify appropriate moments to prompt the stroke survivor | Caregivers require training on *how* to support stroke survivors (i.e. tone – encouraging not nagging, and phrasing /framing e.g. as joint activity) and how often / identifying appropriate moments to prompt | Yes / relevant |
|  | Memory, attention and decision processes | - | - | No / not relevant |
|  | Behavioural regulation | - | Caregivers require strategies to regulate / monitor their prompting, as can forget, and / or suggestions to offer the stroke survivor to monitor their own behaviour, e.g. setting a timer or an alarm | Yes / relevant |
| Physical opportunity | Environmental context and resources | Some caregivers receive support from therapists  Some caregivers live nearby to stroke survivor and personal circumstances (e.g. work, household) permit flexibility when they can see / speak to stroke survivor | Some caregivers do not have contact with therapists – do not receive support offered – support from therapists would increase skill / knowledge / confidence in supporting stroke survivor. For those that cannot get to therapy sessions, there could be an agency / support phone number or online resource to ask questions, and/ or a video.  Personal circumstances of caregiver – work, family responsibilities, location etc. can restrict how often, how much time, and how flexible can be, in seeing / speaking to the stroke survivor. Visiting hours restrict in the inpatient setting. If not possible to have more frequent contact, **or** if the stroke survivor does not have a network of friends/family:   - need support from other family / friends or more formal caregivers (volunteer / befriender / home care etc.) - need to be aware of external groups / services the stroke survivor can access   Adaptations to the home environment (e.g. lowering kitchen surfaces) may make it easier for caregivers to support stroke survivors | Yes / relevant |
| Social opportunity | Social influences | Support from family with other responsibilities enables caregivers / increases caregiver capacity to support the stroke survivor | Social norms relating to slowing down in older age can reduce acceptability of supporting stroke survivor – requires education / clarity re. SB in older age  Some stroke survivors don’t have back-up support from friends and family, or from the stroke survivor themselves (i.e. they don’t want to take part) – would reduce acceptability – need to educate stroke survivor on the importance to reduce their reticence |  |
| Reflective motivation | Professional/social role and identity | Attitudes, behaviour and habits of caregiver being consistent with the aim of the intervention | Attitudes, behaviour and habits of caregiver being inconsistent with the aim of the intervention – for some, change in attitude and SB of caregiver required before can prompt stroke survivor to change  Attitudes towards household roles e.g. who is the cook, can reduce acceptability of certain opportunities such as cooking – need to ensure intervention offers solutions that fit with existing roles | Yes / relevant |
|  | Beliefs about capabilities | Some caregivers are confident in supporting movement as receive training and support from stroke service staff | Stroke survivors’ physical and cognitive impairments, and mood / outlook, can reduce caregivers’ perception of their capability. Require training / support to increase beliefs about capabilities. | Yes / relevant |
|  | Optimism | - | - | No / not relevant |
|  | Beliefs about consequences | - | Caregivers perceive negative consequences outweigh the positive – safety concerns (falls), should be ‘winding down’, lack of understanding of health benefits, don’t want to impact negatively on relationship with caregiver via ‘nagging’ | Yes / relevant |
|  | Intentions | - | - | No / not relevant |
|  | Goals | - | Caregivers don’t currently set goals related to supporting stroke survivors and / or support stroke survivors to set goals – doing so would be helpful | Yes / relevant |
| Automatic motivation | Reinforcement | - | Caregivers often lack support / reinforcement from staff – staff incentivising or reinforcing would be helpful for caregivers | Yes / relevant |
|  | Emotion | - | Caregivers can feel anxious / afraid about encouraging standing due to concerns about falls, especially if the stroke survivor is alone– a careline pendant or similar might reduce feelings of anxiety | Yes / relevant |
